# Unusual case of adult-onset cutaneous lupus erythematosus mimicking early mycosis fungoides

**DOI:** 10.1093/omcr/omad043

**Published:** 2023-05-30

**Authors:** Hanof Ahmed, Mahir Petkar, Omar Wafi, Samya Abu Shaikha

**Affiliations:** Department of Dermatology & Venereology, Hamad Medical Corporation, Doha, Qatar; Translational Research Institute, Hamad Medical Corporation, Doha, Qatar; Weill Cornell Medicine-Qatar, School of Medicine, Doha, Qatar; Department of Health and Life Sciences, King’s College London, London, UK; Department of Pathology, Hamad Medical Corporation, Doha, Qatar; Department of Medicine, Hamad Medical Corporation, Doha, Qatar; Department of Dermatology & Venereology, Hamad Medical Corporation, Doha, Qatar; Translational Research Institute, Hamad Medical Corporation, Doha, Qatar

## Abstract

Discoid lupus erythematosus (DLE) is a chronic variant of cutaneous lupus erythematous developing on sun-exposed areas in multi-morphic forms making diagnosis challenging. Clinical suspicion and prompt treatment are necessary to avoid permanent disfigurement, progression to systemic involvement and poor quality of life. We report a case of delayed DLE diagnosis in a 45-year-old man who presented with a new skin lesion mimicking the early stages of mycosis fungoides that prompted further investigation. Histopathological examination confirmed DLE and appropriate treatment was initiated. However, the atypical clinical presentation led to disseminated DLE and refractory disease control, resulting in scarring and cosmetic disfigurement.

## INTRODUCTION

Lupus erythematosus (LE) is an autoimmune disease existing on a spectrum of cutaneous and systemic manifestations. Discoid lupus erythematosus (DLE) is the most common variant of chronic cutaneous LE that can present in a variety of morphologies [1]. Understanding atypical DLE presentations can aid in early diagnosis and prompt treatment. A clinical diagnosis can only be made with high clinical suspicion and confirmed with histopathological examination. In this case, a patient with erythematous plaques over the scalp developed a new skin lesion on the left upper chest with central clearing and violaceous, erythematous scaly borders, mimicking the early plaque-stage of mycosis fungoides (MF) [2]. Thus, understanding clinical variations in cutaneous presentations of LE can aid in early clinical and confirmatory histopathological diagnosis. Patient outcome depends on high clinical suspicion and prompt treatment to control disease progression and avoid detrimental sequelae.

## CASE REPORT

**Figure 1:**
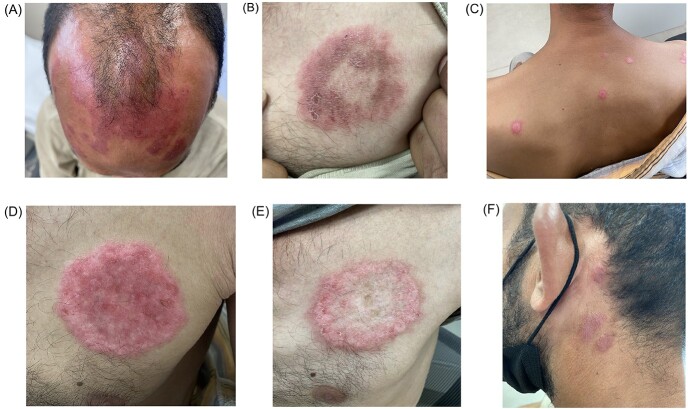
(**A**) Ill-defined irregularly shaped, confluent, erythematous and violaceous plaque on the scalp and upper forehead. (**B**) Pre-treatment lesion showing sharply demarcated erythematous plaque with central clearing and dusky, violaceous and hyperpigmented borders on the left upper chest with an overlying scaly crust. (**C**) Post-treatment new, well-demarcated, erythematous plaques with pigmented borders scattered on the upper back. (**D**) Post-treatment well-defined, infiltrated and brightly erythematous, circular plaque with overlying fine scales on the left upper chest. (**E**) Post-treatment healed circular plaque with erythematous borders and central dyspigmentation (hypopigmentation and hyperpigmentation), atrophy and scarring on the left upper chest. (**F**) Pre-treatment erythematous shiny patches behind the ears.

A 45-year-old male presented with a new erythematous plaque on the left upper chest after an initial diagnosis of photodermatitis due to the presence of erythematous patches and plaques on the scalp (Fig. 1A). The new skin lesion on the left upper chest had raised dusky, violaceous and erythematous borders with central clearing and overlying scaly crusts (Fig. 1B). Erythematous plaques were present behind both ears bilaterally (Fig. 1F). No sensory loss was elicited over the lesions. They were non-painful and non-pruritic. He did not have any systemic symptoms. He reported history of improvement on oral steroids. There was no family history of autoimmune diseases. There was no palpable cervical or axillary lymphadenopathy. Other cutaneous examinations of hair and nails were unremarkable.

**Figure 2:**
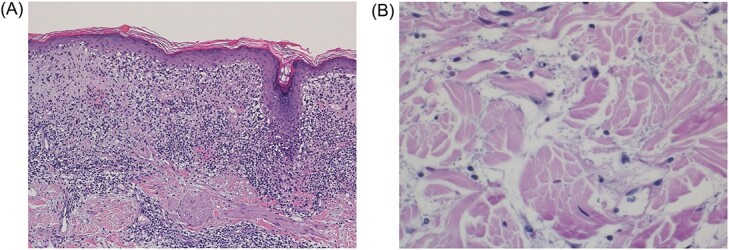
(**A**) Mildly atrophic epidermis with prominent basal layer degeneration and dense lichenoid type of chronic inflammatory infiltrate. Periadnexal and perivascular chronic inflammation are also noted (H and E ×20). (**B**) Interstitial mucin, highlighted by alcian blue stain.

A 4-millimeter punch biopsy from lesional skin showed an orthokeratotic and mildly atrophic epidermis with extensive vacuolar degeneration of the basal layer. There was a dense superficial dermal lichenoid-type of lymphocytic inflammatory infiltrate with scattered melanophages and presence of colloid bodies (Fig. 2A). Marked perieccrine chronic inflammation was noted, extending into the deep dermis. Minimal interstitial chronic inflammation, along with dermal mucin, was present (Fig. 2B). Complete blood count and comprehensive metabolic panel were normal. Antinuclear antibody and anti-Ro were positive with speckled titer pattern. Normal urine protein. Differential diagnosis included MF, other lymphoproliferative disorders of the skin, tuberculoid leprosy and DLE.

Histopathological findings coupled with serological findings were corroborative of DLE and thus the patient was started on short-term oral steroids with prednisolone (20 mg daily), oral hydroxychloroquine (HCQ) (200 mg, twice daily) and high-potency topical steroid clobetasol propionate. Treatment included photoprotection recommendations.

At a 6-week follow-up, new cutaneous lesions appeared on the back (Fig. 1C) and the upper chest lesion became infiltrative (Fig. 1D). The new lesions indicated active disease, and the secondary steroid-sparing immunosuppressant methotrexate (MTX) 15 mg was started with folic acid 5 mg weekly. The patient had weight loss and fatigue, and his daily activities were difficult. In addition, avoidance of sun exposure was challenging due to his occupation and non-adherence to sun protective measures. At 12 weeks, due to the persistence of new lesions, MTX was increased to 20 mg weekly and combined with intralesional corticosteroids with triamcinolone acentonide (40 mg diluted in normal saline and xylocaine 1%, 1:2:1) injected into all cutaneous lesions on the body. The upper chest lesion exhibited signs of healing with presence of residual scar tissue (Fig. 1E). The patient was experiencing discomfort and pain in all new cutaneous lesions.

Patient’s systemic evaluation was negative to date. The patient is under regular follow-up with dermatology and rheumatology.

## DISCUSSION

DLE renders difficulty in clinical suspicion and definitive diagnosis as skin lesions resemble common dermatoses. It can lead to permanent disfigurement with hair loss, dyspigmentation and scarring if not treated early or adequately controlled. The chronic nature of the disease imposes a psychological burden and high morbidity on patients, often requiring psychosocial support [3, 4]. Diagnosis relies on clinical suspicion, and the diagnosis is confirmed by histopathology. Progression to systemic LE differs by subtype, which is higher in generalized DLE (15–28%) than localized DLE (5–10%) [5]. DLE was reported in the literature to mimic a number of diseases including polymorphic light eruption [6], tinea faciei [7], vitiligo [8] and lupus vulgaris [9]. Early diagnosis and treatment remain key to preventing detrimental patient outcomes. In our case, the appearance of a new scaly erythematous plaque in a photoprotected area with hyperpigmented borders, mimicking the patch- or plaque-stage of MF, prompted histopathological evaluation that confirmed DLE [10]. The patient was started on oral steroids, oral HCQ 200 mg twice daily along with high potency topical steroids. The initial misdiagnosis and delayed initiation of DLE treatment resulted in poor disease control, and second-line oral MTX 15 mg weekly with folic acid 5 mg was added and increased to 20 mg weekly due to progressively new skin lesions. Intralesional steroid injections were added as an alternative local therapy. This patient’s progression to generalized DLE increases the risk of systemic involvement. Multiple treatment modalities were initiated in effort to control the appearance of new skin lesions and further disfigurement. In other cases of insufficient response to HCQ, a cohort of patients showed response to chloroquine or even combination therapy. However, no new lesions appeared and infiltrative lesions showed gradual improvement on subsequent follow-up, healing with surrounding hyperpigmentation and central scarring, hypopigmentation and atrophy. To avoid detrimental sequelae of DLE, early diagnostic evaluation with histopathological examination and prompt treatment is necessary to halt progression of disease towards permanent disfigurement and progression to systemic LE.

## Data Availability

Data resulted from this study are available from the corresponding author on reasonable request.
